# *Schistosoma Mansoni* and *Haematobium*: Radiological Diagnostic Clues and Pathophysiology

**DOI:** 10.3390/pathogens15050536

**Published:** 2026-05-15

**Authors:** Sultan Abdulwadoud Alshoabi, Abdullatif O. Magram, Abdulaziz H. Alkalady, Rafat Rashed Al-Maqtari, Khaled M. Almas, Khaled Mohammed Al-Sayaghi, Abdullgabbar M. Hamid, Fahad H. Alhazmi, Abdulaziz A. Qurashi, Walaa Alsharif, Amirah Alsaedi, Ezzat AbuAzzah, Abdulkareem Algahtani, Khaled A. Alqfail, Khalid M. Alshamrani

**Affiliations:** 1 Department of Diagnostic Radiology, College of Applied Medical Sciences, Taibah University, Al-Madinah Al-Munawwarah 42353, Saudi Arabia; 2 Advanced AlRazi Diagnostic Center, Al-Hodeidah, Yemen; 3 Berlin Scan Center, Ibb, Yemen; 4 Department of Medical Surgical Nursing, College of Nursing, Taibah University, Al-Madinah Al-Munawwarah 42353, Saudi Arabia; 5 Radiology Department, Rush University Medical Group, Chicago, IL 60612, USA; 6 Department of Radiology, College of Medicine, Najran University, Najran P.O. Box 1988, Saudi Arabia; 7 College of Applied Medical Sciences, King Saud bin Abdulaziz University for Health Sciences, Jeddah 22384, Saudi Arabia; 8 King Abdullah International Medical Research Center, Jeddah 22384, Saudi Arabia; 9 Ministry of the National Guard—Health Affairs, Jeddah 22384, Saudi Arabia

**Keywords:** hepatic Schistosomiasis, genitourinary Schistosomiasis, periportal fibrosis, liver cirrhosis, intestinal polyposis, urinary bladder calcification, seminal vesicle calcification

## Abstract

Schistosomiasis (bilharzia) is a parasitic infection caused by trematodes of the *Schistosoma* genus and remains a significant health burden in endemic regions. Granulomatous host responses to deposited *Schistosoma* eggs in small veins and tissues result in progressive changes and characteristic imaging findings. This diagnostic radiological review synthesizes the published literature and highlights key and robust imaging findings that facilitate the diagnosis of *Schistosoma mansoni* and *Schistosoma haematobium*, with emphasis on modality-specific patterns and disease staging. *Schistosoma mansoni* primarily affects the liver, causing periportal fibrosis visible as “pipe-stem” echogenic thickening upon ultrasonography, which may progress to portal hypertension and chronic liver disease. Liver cirrhosis is the end-stage disease manifested as an irregular liver contour with surface nodularity and lobar redistribution as right lobe atrophy with left and/or caudate lobe hypertrophy. *Schistosoma haematobium* predominantly affects the genitourinary system, causing urinary bladder wall thickening and calcification. Early disease, within three months of infection, may present with fine calcification, firstly in the bladder base and then extending to the whole bladder and even to the ureters. Calcification appears as a line or two parallel lines on radiography and as a circle in axial computed tomography (CT) images, which is pathognomonic for early-stage Schistosomiasis. In contrast studies, including conventional urography and CT urography, *Schistosoma* eggs appear as bubble-like filling defects in the ureter, kidney, and bladder, manifested as ureteritis, pyelitis, and cystitis cystica. Late stages appear as coarse calcification, fibrosis, strictures, and reduced bladder capacity and are associated with an increased risk of bladder squamous cell carcinoma. Moreover, Schistosomiasis calcification can present in genital organs, especially in the seminal vesicles; in the prostate in males; and in the vulva, cervix, and perineum in females. Ultimately, *Schistosoma mansoni* and *haematobium* eggs can reach the spinal cord, leading to acute myelopathy with paraparesis, urinary retention, or paraplegia. Recognition of characteristic imaging patterns of Schistosomiasis is essential for early diagnosis, accurate staging, and prevention of long-term complications.

## 1. Introduction

Schistosomiasis, also known as bilharzia, is a neglected parasitic infection caused by a flatworm of the *Schistosoma* genus [1]. Schistosomiasis is the second most common pathogenic parasitic infection after malaria, affecting people in many low- and middle-income countries; thus, it has been targeted for elimination by the World Health Organization (WHO) by 2030 [2]. According to the WHO, *Schistosoma* affects 240,000,000 people across 78 countries, over 90% of whom live in Africa, and many individuals required preventive chemical therapy for Schistosomiasis in 2020 [3].

In humans, Schistosomiasis is caused by five main pathogenic parasitic species: *Schistosoma mansoni*, *Schistosoma japonicum*, *Schistosoma haematobium*, *Schistosoma intercalatum*, and *Schistosoma mecongi*. The first three are the most common species. *Schistosoma mansoni* is prevalent in Africa, the Middle East, the Caribbean, and South America. *Schistosoma japonicum* is found in China, the Philippines, and the Indonesian island of Sulawesi. *Schistosoma haematobium* is endemic in Africa and the Middle East [4]. *Schistosoma* is transmitted to humans through direct contact with freshwater contaminated with cercariae that can penetrate the skin [5].

Medical imaging modalities have an essential role in directing the diagnosis of Schistosomiasis through highly suggestive medical imaging features. Ultrasonography is considered a non-invasive, radiation-free, and highly effective key imaging modality in detecting structural damage caused by Schistosomiasis, such as periportal fibrosis and signs of portal hypertension in *Schistosoma mansoni* [6] and urinary bladder wall thickening and lesions in *Schistosoma haematobium* [7]. However, ultrasonography is limited by operator-dependent accuracy and artifact pitfalls, especially in the hepatobiliary system [8]. Computed tomography (CT) is a highly valuable imaging modality offering superior accuracy in detecting chronic fibrosis, calcification patterns, and even ectopic lesions such as brain and spinal Schistosomiasis [6]. However, CT is limited by high radiation exposure, risk of iodinated contrast agents, cost and accessibility, and low sensitivity in early disease and active infection [9]. Magnetic resonance imaging (MRI) is a powerful, radiation-free imaging modality for the diagnosis of Schistosomiasis that can cover central nervous system Schistosomiasis and advanced liver fibrosis with the offer of superior tissue characterization compared to ultrasonography and CT. However, MRI is limited due to its high cost, poor availability in endemic regions, and difficulty in detecting small calcifications [9].

This study aims to review radiological pictorial images that can provide robust diagnostic clues of *Schistosoma*. This pictorial review will help radiologists, urologists, and internal medicine physicians who are interested in the diagnosis of *Schistosoma*.

## 2. Methods

This is a pictorial radiological review focusing on the imaging features of Schistosomiasis. It evaluates characteristic radiological findings reported in the literature, complemented by representative cases obtained from our diagnostic center database. Radiological images were retrospectively retrieved from cases diagnosed at the Advanced AlRazi Diagnostic Center, Al-Hodeidah, Republic of Yemen. Ultrasonography, CT, and MRI images demonstrating significant classical features of Schistosomiasis were selected. All images were evaluated by two experienced radiologists to ensure diagnostic accuracy and optimal image quality. Images were selected based on their ability to clearly demonstrate key diagnostic features, including hepatic periportal fibrosis, portal hypertension, urinary bladder wall thickening, calcifications, and other organ-specific manifestations. This pictorial review emphasizes the correlation between radiological findings and underlying pathology, aiming to provide clinicians, including radiologists, urologists, infectious disease specialists, and internal medicine physicians, with practical visual references to support accurate and timely diagnosis. A targeted literature review was conducted using the major medical database PubMed. Article selection was guided by the relevance of the study to Schistosomiasis imaging features, their diagnostic significance, and pathophysiology.

## 3. Life Cycle of *Schistosoma*

Infected mammalian hosts, including humans, mice, or dogs, release eggs through faeces (*Schistosoma mansoni*) or urine (*Schistosoma haematobium*). In fresh water, these eggs develop into miracidia, which infect snails (intermediate host). In infected snails, miracidia transform into mother sporocysts, generating multiple secondary sporocysts and ultimately developing into multiple cercariae. Cercariae can penetrate the epidermis of humans or another mammal (definitive host). In the skin, cercariae then transform into schistosomula. Schistosomula migrate through the dermis to the dermal blood vessels. Approximately three days postinfection, *Schistosoma mansoni* and *Schistosoma haematobium* leave the blood vessels and reach the lung through the right side of the heart and pulmonary artery. They can flow within the blood via the pulmonary veins to the left side of the heart and can be distributed to the different organs of the body [10].

**Figure 1:**
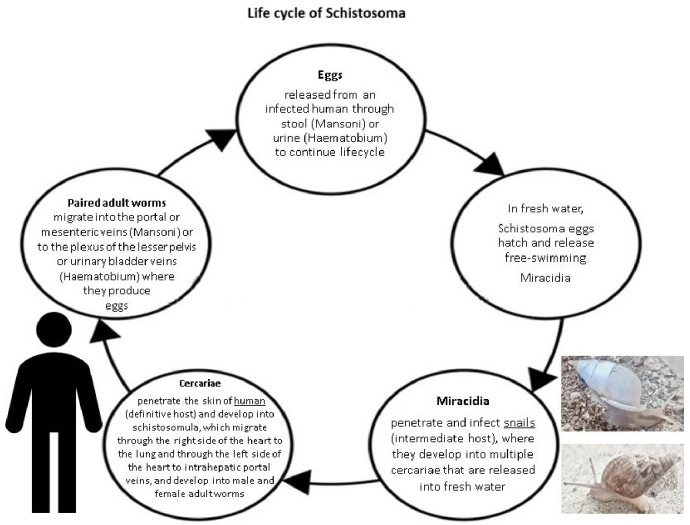
Lifecycle of *Schistosoma*.

In human intrahepatic portal veins, schistosomula develop into male and female worms. Adult *Schistosoma* worms typically reside in pairs (male and female) within the lumen of the blood vessels. Mature *Schistosoma* worms then migrate through blood vessels to small mesenteric veins (*Schistosoma mansoni*) or to the plexus of the lesser pelvis or urinary bladder (*Schistosoma haematobium*), where they lay eggs. These eggs accumulate in the vascular lumen and trigger an inflammatory response, forming granulomas that help them pass through soft tissue into the lumen of the intestine, urinary bladder, or ureter, where they are excreted through stool or urine [11,12] (Figure 1).

## 4. Pathophysiology of Schistosomiasis

Schistosomiasis pathogenesis is primarily caused by the immune response of the infected human to the *Schistosoma* eggs that are trapped in the small veins and tissues, causing granulomatous inflammatory reactions [13]. The adult parasites of *Schistosoma mansoni* establish themselves in the lower mesenteric veins, where they lay approximately 200–300 eggs daily. The non-excreted eggs in faeces cause granulomatous inflammatory disease [4]. The adult worm of *Schistosoma japonicum* primarily inhabits the superior mesenteric veins, where it lays eggs that can easily reach the liver or intestine, leading to rapid progression to liver cirrhosis and potentially liver failure [4]. The adult female of *Schistosoma haematobium* lays eggs in the veins of the main organs of the pelvis and is responsible for urogenital pathology. The International Agency for Research on Cancer has classified Schistosomiasis as a probable human carcinogen that can cause squamous cell carcinoma or urothelial carcinoma of the urinary bladder [4,14].

## 5. Diagnosis of *Schistosoma*

**Figure 2:**
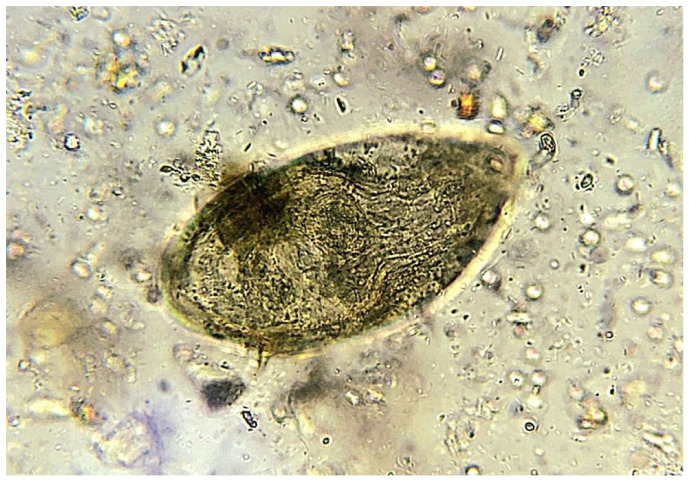
Egg of *Schistosoma mansoni* with a prominent lateral spine appears under microscopic examination of a stool sample with a high-power lens (40×) with no stain.

**Figure 3:**
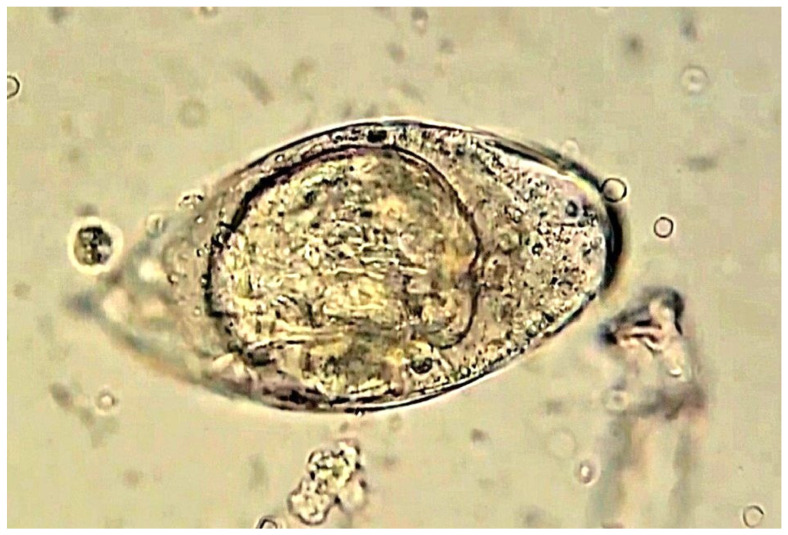
Egg of *Schistosoma haematobium* with prominent terminal spine appears under microscopic examination of a urine sample with a high-power lens (40×) with no stain.

Accurate diagnosis is essential for the surveillance and control of Schistosomiasis. Detecting the parasite eggs either in stool (*Schistosoma mansoni*) or in urine (*Schistosoma haematobium*) is considered the gold standard and a WHO-recommended confirmatory diagnostic test [3,15]. In reality, the microscopy-based Kato-Kats technique and urine filtration are the reference diagnostic tests for *Schistosoma mansoni* and *Schistosoma haematobium*, respectively [16]. In addition to microscopy, a range of diagnostic tests is available, including antibody detection, antigen detection using the POC-CCA “Point-Of-Care Circulating Cathodic Antigen”, as well as NAATs (Nucleic Acid Amplification Tests) such as real-time PCR “Polymerase Chain Reaction” [17] (Figure 2 and Figure 3).

## 6. Medical Imaging of *Schistosoma*

Medical imaging modalities, including conventional radiography, ultrasonography, CT, and MRI, play a crucial role in diagnosing Schistosomiasis through detecting organic changes that form robust diagnostic clues. In addition, they may be helpful in evaluating disease severity and complications [9]. This pictorial review concentrated on revising the common radiological clues leading to *Schistosoma* diagnoses.

### 6.1. Medical Imaging Clues in Hepatointestinal Schistosomiasis

Hepatic Schistosomiasis results from a heavy *Schistosoma mansoni* infection of the liver, driven by the host’s cell-mediated immune response to soluble egg antigens, which can progress to irreversible fibrosis and consequently portal hypertension. *Schistosoma mansoni* eggs enter the liver and, after three weeks, cause a moderate type 1 (Th1) response to egg antigens. This response usually progresses to a dominant Th2 immune response to egg-derived antigens with subsequent recruitment of eosinophils and granuloma formation to block the hepatotoxic effect of the antigens released from the eggs. Due to inflammatory reactions to the parasitic eggs trapped in liver sinusoids with the consequent deposition of collagen and extracellular matrix proteins in the periportal space, periportal fibrosis and portal hypertension represent the key radiological hallmarks of Schistosomiasis [18,19]. Periportal fibrosis, one of the major complications of Schistosomiasis, appears as echogenic thickening of the wall of the portal vein and its branches—described as “Symmers’ pipe-stem fibrosis” [20].

Liver cirrhosis is irreversible architectural damage, which is the endpoint of chronic liver disease and is the most common cause of portal hypertension and the greatest risk of hepatocellular carcinoma [21]. The main ultrasonography signs of liver cirrhosis are irregular liver contour and surface nodularity due to fibrotic changes and regenerative nodules, thick and heterogeneous echogenicity of the liver, and abnormal liver proportions, including right lobe atrophy and left and/or caudate lobe hypertrophy [22,23]. Portal hypertension is manifested as portal vein dilatation (≥13 mm), with ascites (up to 70%) and/or hydrothorax (up to 15%); splenomegaly (spleen more than 13 cm in craniocaudal diameter); portosystemic shunts, such as gastroesophageal varices and paraoesophageal varices; recanalized umbilical veins with dilated paraumbilical veins; splenorenal shunts; gastrorenal shunts; mesocaval shunts; peristomal varices; and perirectal varices. On spectral Doppler ultrasound, portal vein velocities less than 20 cm/s are diagnostic of portal hypertension with 80% sensitivity and specificity. Reversal flow of the portal vein is pathognomonic of portal hypertension [23].

**Figure 4:**
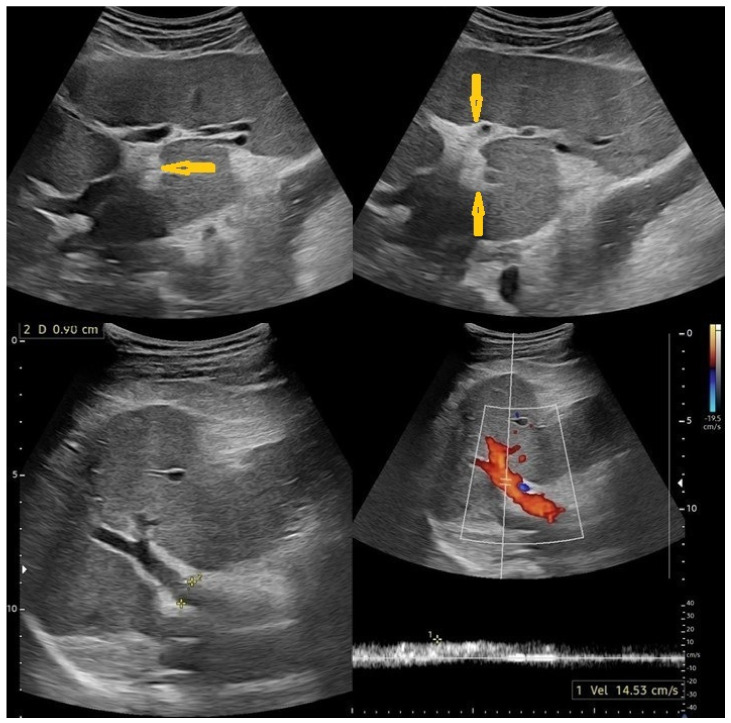
Selected sections of ultrasonography of a 40-year-old man’s liver show periportal fibrosis (arrows) of hepatic Schistosomiasis with an average liver size and normal echo patterns, no focal lesion, portal vein (PV) diameter = 9 mm with reduced flow velocity to 14 cm/s, and no prominent portosystemic collateral.

**Figure 5:**
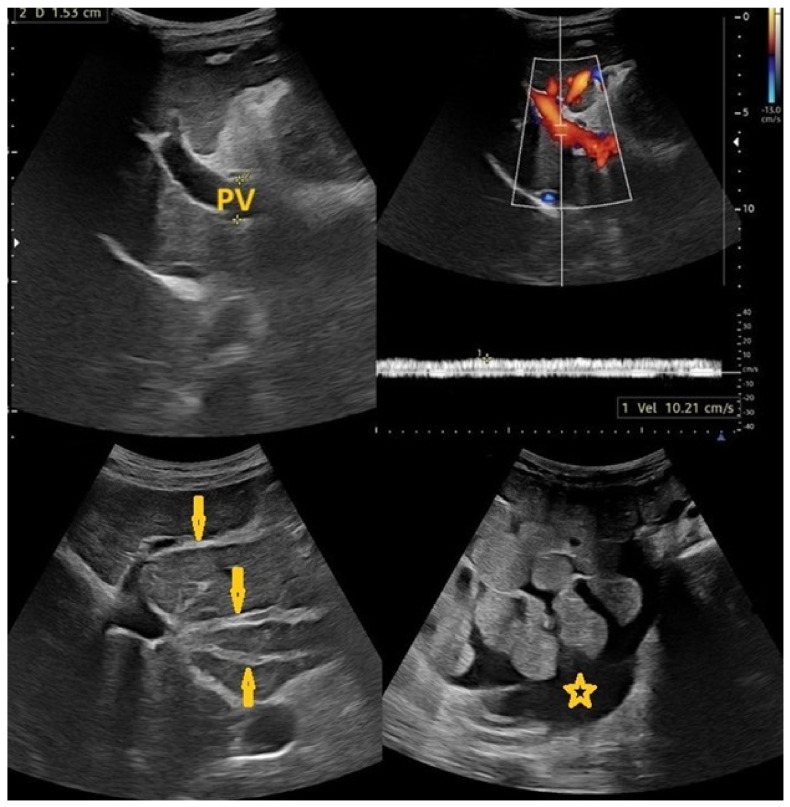
Selected sections of ultrasonography of a 32-year-old woman’s abdomen show an average-size liver with irregular borders, coarse texture, periportal fibrosis (arrows), and signs of portal hypertension, including the following: ascites (star) and splenomegaly (up to 15 cm in craniocaudal diameter, portal vein diameter of 14 mm, hepatofugal flow with reduced velocity in PV to 12 cm/s, with portosystemic collateral veins in the epigastric and splenic hila). Picture of liver cirrhosis with portal hypertension and complicated chronic Schistosomiasis.

**Figure 6:**
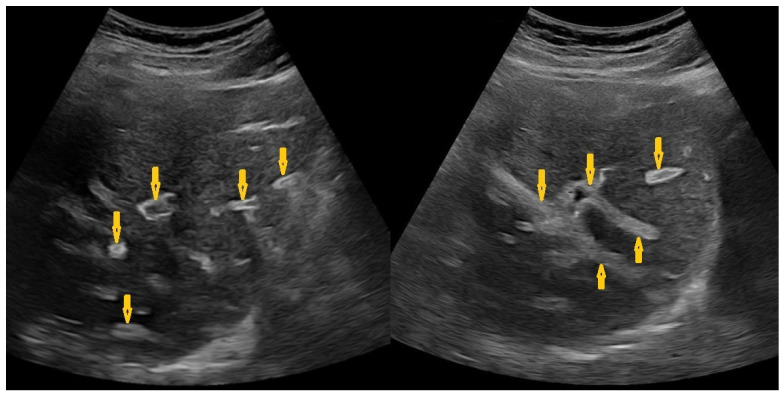
Selected sections of ultrasonography of a 79-year-old man’s liver show irregular borders, coarse texture, periportal fibrosis (arrows), and signs of portal hypertension, including mild splenomegaly. Picture of chronic liver disease with portal hypertension as a result of chronic Schistosomiasis.

**Figure 7:**
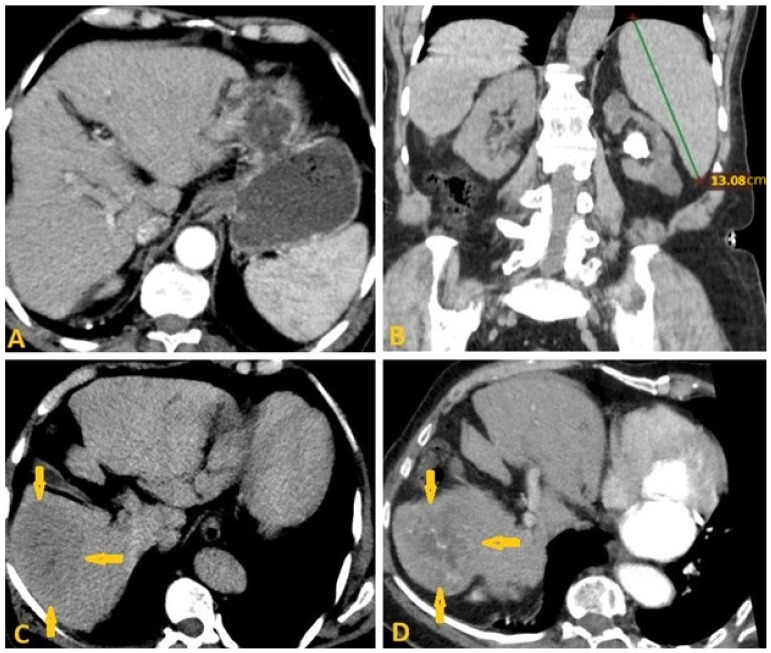
Selected computed tomography images (same patient as in Figure 6: axial (**A**,**C**,**D**) and coronal reconstruction (**B**) of a 79-year-old man show irregular borders of the liver with widening interlobar fissure in keeping with chronic liver disease (**A**,**B**). In addition, there is a 75 × 57 mm ill-defined hypodense mass in segment-6 (arrows on (**C**)), revealing early heterogeneous enhancement after contrast administration (arrows on (**D**)) that rapidly washed out in the delayed phase, which is consistent with hepatocellular carcinoma complicated by liver cirrhosis as a result of chronic Schistosomiasis.

In the small intestine, egg-laying worms are present in the intestinal microvasculature in the distribution of the inferior mesenteric venous plexus. In the large intestine, eggs are mainly distributed in the submucosa and subserosa, where multiple granulomas are frequently formed. Subsequently, the muscularis mucosa and the overlying mucosa either form superficial ulcers or undergo hyperplastic changes. The submucosa becomes thickened by fibrous tissue containing immense numbers of calcified eggs forming sandy patches, and the overlying mucosa becomes atrophic with a granular, dirty yellowish appearance [19,24]. Polyp formation starts with the deposition of *Schistosoma* eggs in the submucosa, which produces a cell-mediated inflammatory immune response with granuloma formation and necrosis. The necrotic foci heal, and fibrous connective tissue is formed with hypertrophy of the adjacent muscularis mucosa. The fibrous tissue forms a barrier to the usual route of *Schistosoma* ova transit from mesenteric veins to the gut lumen. This entrapment of *Schistosoma* ova leads to a foreign body reaction with progressive inflammation and reaction, and a nodule is formed that elevates the hypertrophied muscularis mucosa and mucosa to form a polyp [19,25]. *Schistosoma mansoni* can cause hepatomegaly, splenomegaly, and intestinal polyposis, and it can occasionally cause colonic polyposis [26,27] (Figure 4, Figure 5, Figure 6 and Figure 7).

### 6.2. Medical Imaging Clues in the Genitourinary Schistosomiasis

Radiological clues can suggest Schistosomiasis even in early stages, within three months of *Schistosoma haematobium* infection. Fine ureteral calcification that appears as a line or two parallel lines on conventional abdominopelvic radiography and as a circular pattern on axial CT images is pathognomonic for early-stage Schistosomiasis. In cases of ureteritis, pyelitis, and cystitis cystica, intravenous urography and intravenous CT urography demonstrate bubble-like filling defects representing *Schistosoma* eggs in the ureter, kidney, and urinary bladder. Coarse calcification, fibrosis, and strictures are signs of late-stage Schistosomiasis. Severe changes in the urinary bladder may predispose to squamous cell carcinoma [28]. *Schistosoma haematobium* is manifested by calcification along the bladder base, extending to include the whole bladder wall [29]. In addition, calcification can be present in ureteric walls; seminal vesicles; prostate in males; and the vulva, cervix, and perineum in females [28,30]. However, we should remember tuberculosis as a differential diagnosis of seminal vesicle calcification [31] and other genitourinary organs [32,33]. Urinary bladder cancer, urease-producing bacterial infection, cytokine-induced inflammatory processes, and coronavirus disease 2019 are reported causes of urinary bladder wall calcification [34].

**Figure 8:**
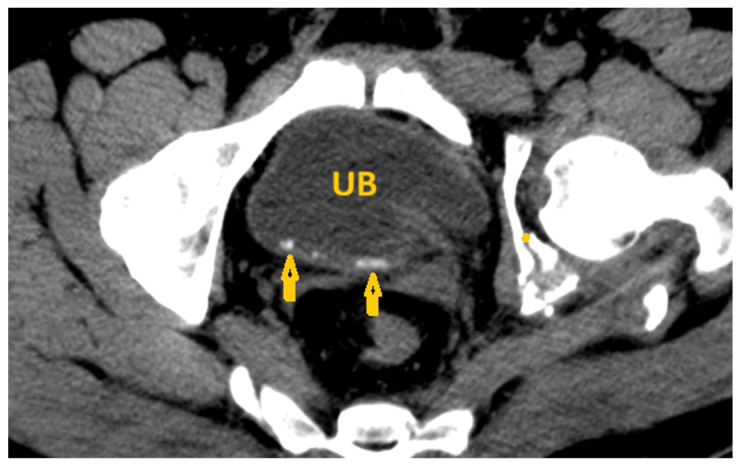
Selected non-contrast computed tomography axial pelvic image of a 36-year-old male patient shows a small linear calcification at the base of the urinary bladder (arrows), reflecting *Schistosoma* cystitis. UB: Urinary bladder.

**Figure 9:**
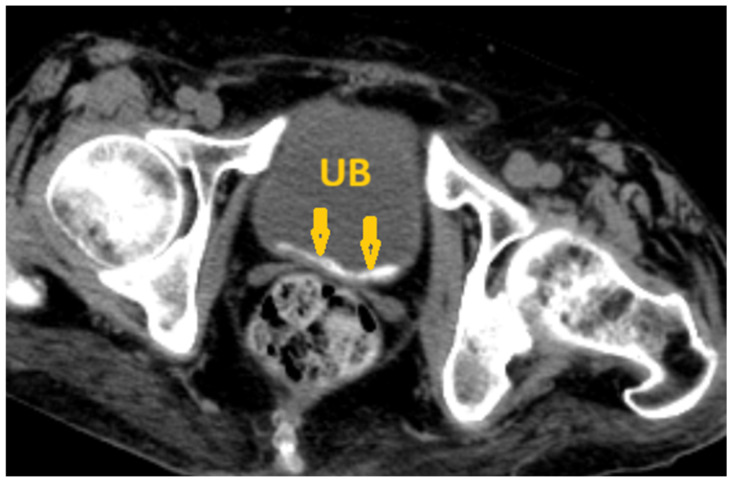
Selected non-contrast computed tomography axial image of a 40-year-old male patient shows linear calcification at the posterior wall of the urinary bladder (arrows), reflecting *Schistosoma* cystitis. UB: Urinary bladder.

**Figure 10:**
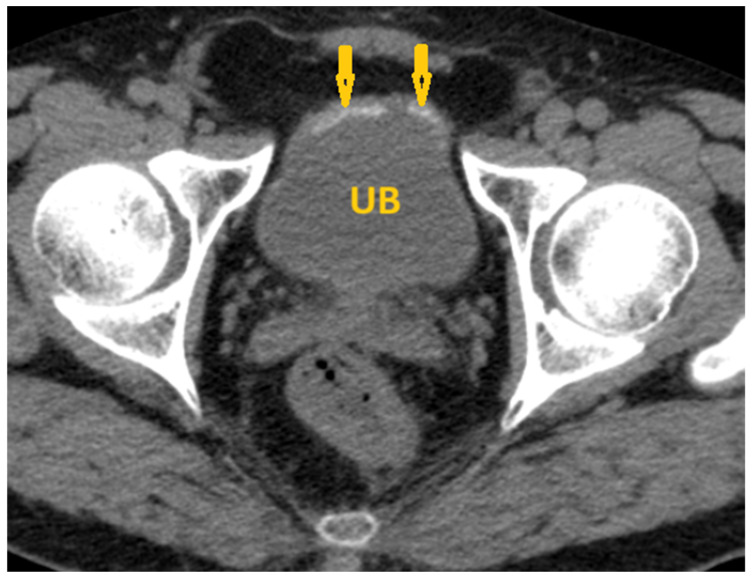
Selected non-contrast computed tomography axial image of a 46-year-old male patient shows mildly thickened urinary bladder wall with linear calcification (arrows) at the anterior aspect, reflecting *Schistosoma* cystitis. UB: Urinary bladder.

**Figure 11:**
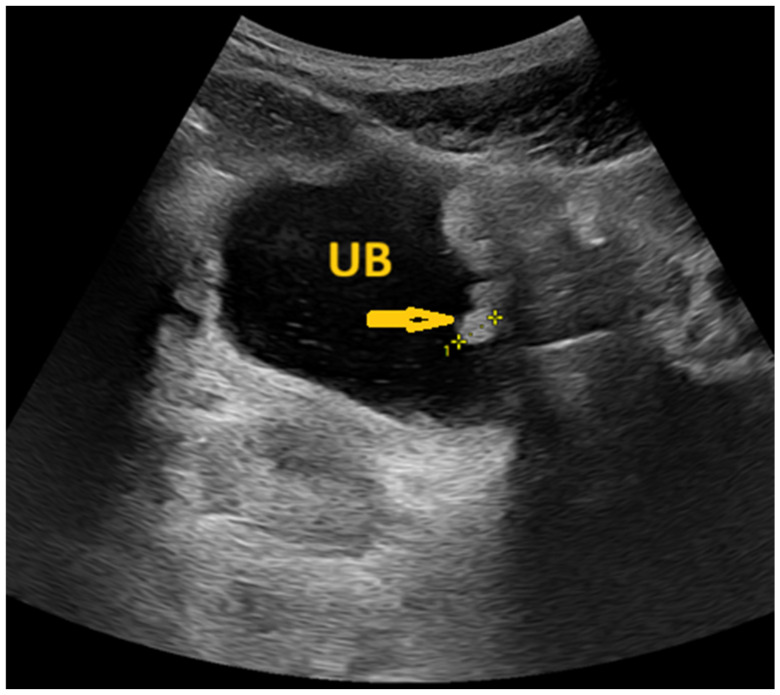
Selected ultrasonography image of a 20-year-old male patient shows multiple polypoidal thickening in the left urinary bladder wall, the largest measuring 10 × 5 mm (arrow), reflecting *Schistosoma* cystitis. UB: Urinary bladder.

**Figure 12:**
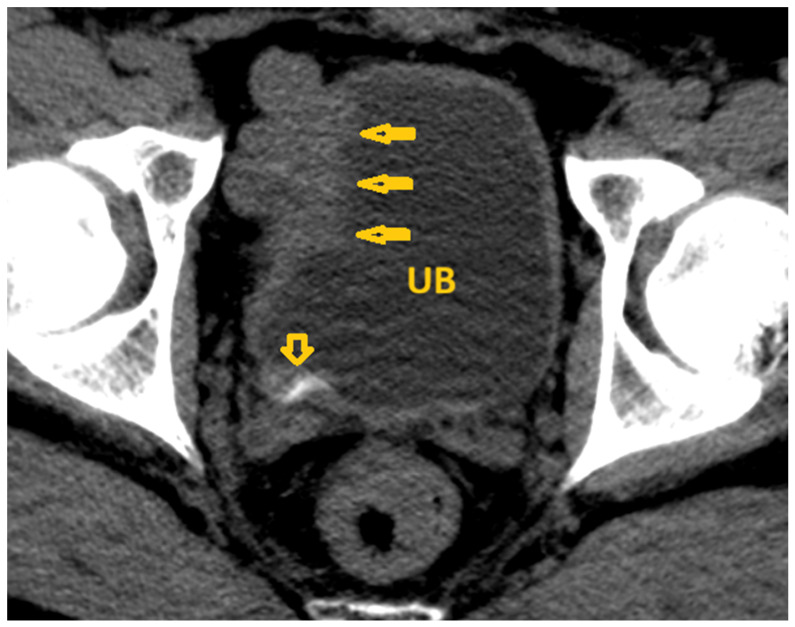
Selected non-contrast computed tomography axial image of a 27-year-old male patient shows polypoidal thickening of the right urinary bladder wall (long arrows) with small calcification seen at the right distal ureter, reflecting *Schistosoma* cystitis (short arrow). UB: Urinary bladder.

**Figure 13:**
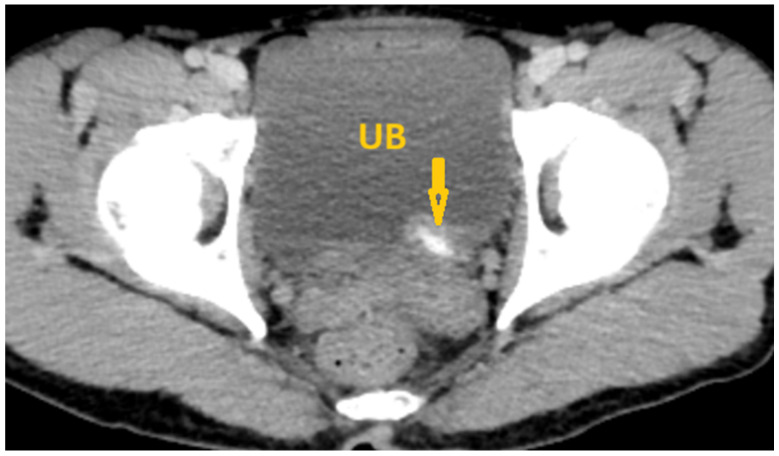
Selected non-contrast computed tomography axial image of a 23-year-old male patient shows a markedly thickened wall of the urinary bladder at the left vesico-ureteric junction with calcification (arrow), causing mild effacement of the lumen and reflecting *Schistosoma* cystitis. UB: Urinary bladder.

**Figure 14:**
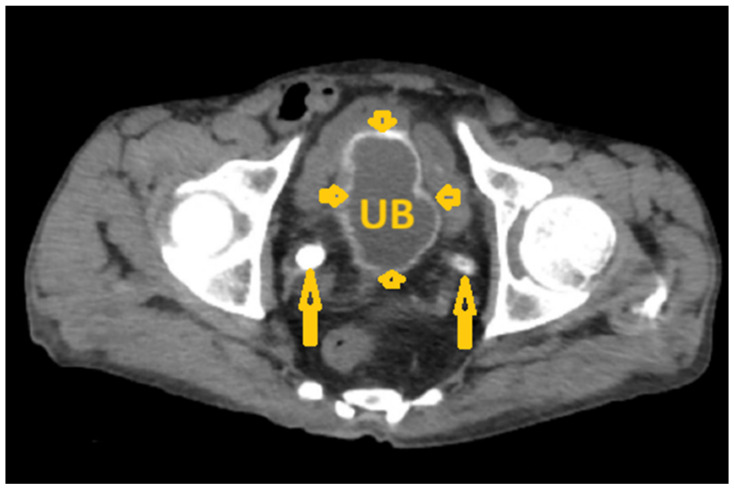
Selected non-contrast computed tomography axial image of a 64-year-old male patient shows diffuse calcification of the urinary bladder wall (short arrows) and bilateral distal ureter calcification (long arrows), reflecting *Schistosoma* cystitis in a proven case of chronic infection with *Schistosoma haematobium*. UB: Urinary bladder.

**Figure 15:**
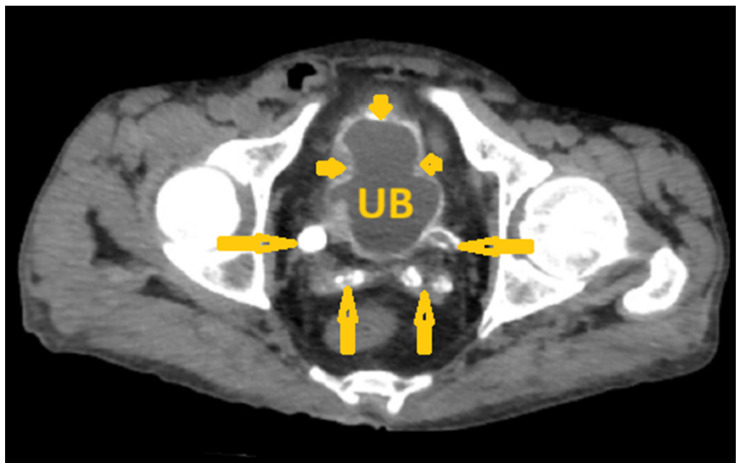
Selected non-contrast computed tomography axial image of a 64-year-old male patient (same patient as in Figure 14) shows diffuse calcification of the urinary bladder wall (short arrows), extending to the bilateral ureterovesical junction (lateral arrows), and also shows bilateral seminal vesicle calcification (posterior arrows), reflecting *Schistosoma* cystitis in a proven case of chronic infection with *Schistosoma haematobium*. UB: Urinary bladder.

**Figure 16:**
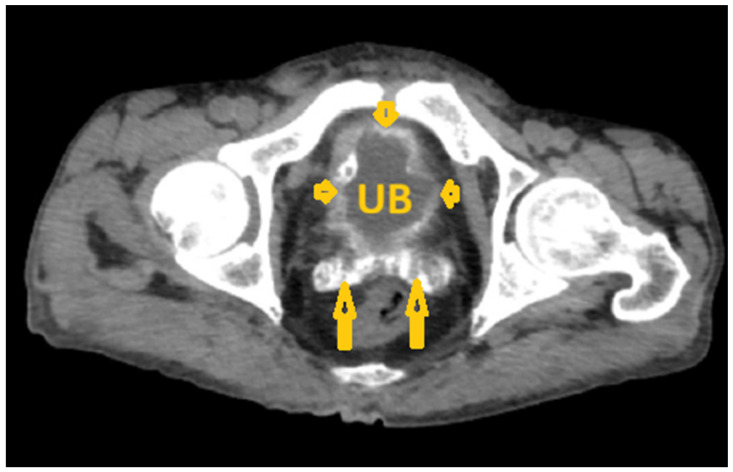
Selected non-contrast computed tomography axial image of a 64-year-old male patient (same patient as in Figure 14 and Figure 15) shows thick urinary bladder wall with circumferential linear mural calcification (short arrows) and bilateral seminal vesicle calcification (long arrows) in a proven case of chronic infection with *Schistosoma haematobium*. UB: Urinary bladder.

**Figure 17:**
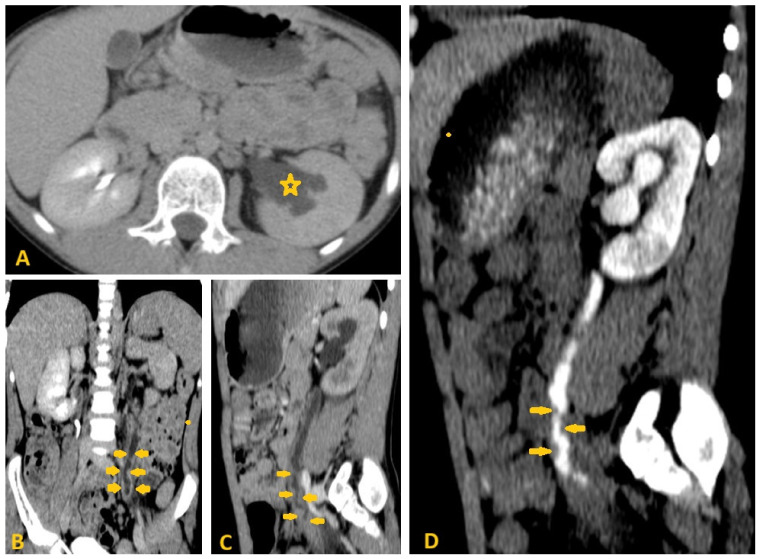
Selected post-contrast computed tomography images of a 10-year-old boy presenting with left abdominal pain show delayed excretion of contrast in the left kidney with moderate left hydroureteronephrosis (star on the axial image (**A**)) due to circumferential wall thickening at the middle part of the left ureter causing severe ureteric liminal narrowing (arrows on coronal reconstruction image (**B**) and sagittal reconstruction image (**C**)). Delayed sagittal reconstruction image (**D**) shows multiple filling defects in the narrowed ureter (arrows). This case was diagnosed as ureteric Schistosomiasis, and follow-up images showed improvement after *Schistosoma* treatment.

**Figure 18:**
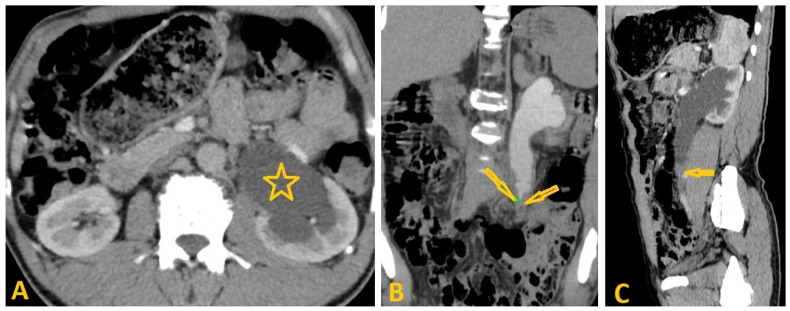
Selected non-contrast computed tomography axial images of a 45-year-old male patient presenting with left abdominal pain show left kidney delayed excretion of contrast for 30 min with moderate left hydroureteronephrosis (star on the axial image (**A**)) due to circumferential ureteral wall thickening at the middle part (arrows on coronal reconstruction image (**B**)), causing severe ureteric liminal narrowing measuring 6 cm in length (arrow on sagittal reconstruction (**C**)). In addition, there is 9 × 4 mm oval-shaped left para-aortic enlarged lymph node. This case was diagnosed as left ureteric transitional cell carcinoma in a proven case of chronic Schistosomiasis.

**Figure 19:**
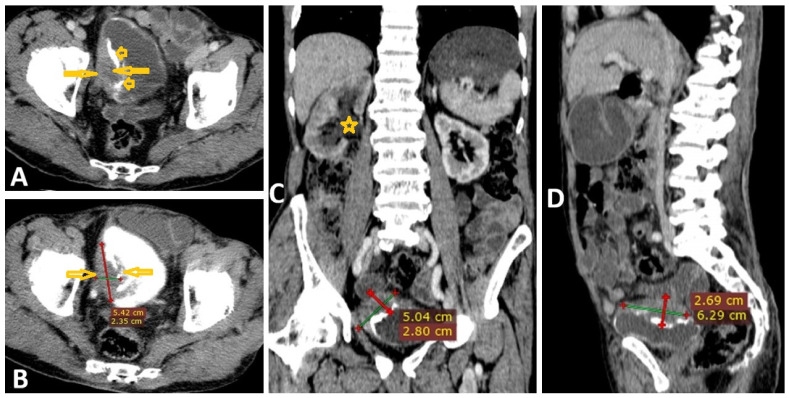
Selected computed tomography (CT) images of an 80-year-old male patient with a known case of chronic infection with *Schistosoma haematobium* who presented with frequent urination. CT images show a heterogeneously enhanced mass (arrow on axial image (**A**)) measuring about 63 × 54.2 × 28 mm infiltrating the right supero-lateral wall of the urinary bladder (UB) with foci of calcification (arrow heads on axial image (**A**)) in the wall of the UB. The mass appears as a filling defect in the UB with contrast axial images (arrows on (**B**)), which infiltrates the right vesico-ureteric junction, causing secondary right mild hydroureteronephrosis (star on coronal reconstruction image (**C**)). Sagittal reconstruction image (**D**) shows that the lesion infiltrates the perivesical fat with no infiltration into the muscle and bone of the pelvic wall, consistent with UB cancer.

Pathophysiology of calcification in the urinary system is primarily due to the human immune response to *Schistosoma haematobium* eggs trapped in the submucosa of the bladder. Dead calcified eggs with granulomas and fibrosis cause patches and linear calcification, resulting in chronic damage, strictures, and increasing risk of squamous cell carcinoma. In females, the human immune response induces granuloma formation around the deposited eggs, resulting in various mucosal changes such as sandy patches, rubbery papules, and angiogenesis, which can be seen using colposcopy. These lesions can induce severe complications, such as increased risk of HIV infection, infertility, ectopic pregnancy, and abortion [35] (Figure 8, Figure 9, Figure 10, Figure 11, Figure 12, Figure 13, Figure 14, Figure 15, Figure 16, Figure 17, Figure 18 and Figure 19).

### 6.3. Medical Imaging Clues in Central Nervous System Schistosomiasis

**Figure 20:**
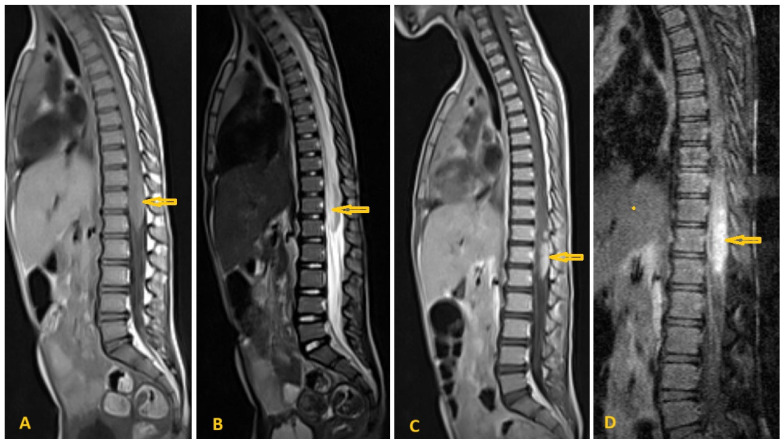
Selected sagittal images of lumbar spine MRI of a 5-year-old girl who presented with lower limb weakness and urine retention for a week and who had a history of excessive swimming in a valley during a journey two months before. The selected images show isointense expansion of the distal spinal cord and conus medullaris (arrows) in the T1-weighted image (**A**), with patchy hyperintense foci in T2-weighted images (**B**), intramedullary peripheral nodular enhancement in Gadolinium-enhanced T1-weighted images (**C**), and intramedullary increased signal intensity in the fat-suppression image (**D**). MRI revealed no extension into the neural foramina and no bone or surrounding soft tissue edema. The whole picture was consistent with inflammatory spinal Schistosomiasis.

Neuroschistosomiasis is a severe complication of *Schistosoma* infection triggered by the immune reaction to *Schistosoma* eggs deposited in the central nervous system, which constitutes less than 4% of cases of Schistosomiasis. *Schistosoma* eggs travel from the hepatic portal veins through the valveless paravertebral veins of Batson (Batson venous plexus) to the lower spinal cord, where adult worms travel to reach the CNS and produce eggs in local venules [36]. *Shistosoma japonicum* usually causes encephalopathy, while *Shistosoma mansoni* and *Shistosoma haematobium* usually cause myelopathy. The clinical manifestations and outcome of the neurological disorder depend on the phase and the clinical form of Schistosomiasis [37]. Cerebral complications are more prevalent than their spinal counterparts. In total, 90% of cases develop neurological deficits within two months of the infection. Seizure and encephalopathy are the most common features [38]. When *Shistosoma mansoni* and *Shistosoma haematobium* eggs are deposited in the spinal cord, they lead to inflammatory immune-mediated disease characterized by intense granulomatous reaction primarily affecting the conus medullaris and lower thoracic spinal cord. Acute myelopathy is a neurological complication of Schistosomiasis presented by paraparesis and urinary retention or paraplegia that may be preceded by lumbar pain for hours to weeks. Investigations include stool and urine analysis, spinal MRI, blood tests, and cerebrospinal fluid analysis. Diagnosis is suggested by clinical features, history, and radiological evidence of active Schistosomiasis. However, definitive diagnosis is achieved by the detection of *Schistosoma* eggs in the spinal cord biopsy [39] (Figure 20).

## 7. Limitations

This review focuses on *Shistosoma mansoni* and *Shistosoma haematobium,* which are the prominent species in the Middle East; *Shistosoma japonicum* is not considered, therefore limiting the generalizability of the findings to regions where this species is endemic. Additionally, radiological illustrations of intestinal manifestations, such as polyps, were not included due to their infrequent detection on routine imaging modalities. Similarly, pulmonary involvement was not illustrated as imaging findings are often non-specific and lack distinctive features that reliably differentiate Schistosomiasis from other etiologies. Radiological images of Schistosomiasis of the female genital organs were not available in our center.

## 8. Conclusions

In infected humans, the immune response to *Schistosoma* eggs trapped in small veins and tissues cause granulomatous inflammatory reactions and changes in the affected organs, providing robust diagnostic clues in radiological images that could result in a reliable diagnosis of *Schistosoma*. *Shistosoma mansoni* can cause periportal fibrosis, portal hypertension, liver cirrhosis, intestinal polyposis, and occasionally colonic polyposis. *Shistosoma haematobium* mainly affects the genitourinary system and is typically manifested as calcification along the urinary bladder base that extends to the whole bladder wall. Radiological clues can suggest Schistosomiasis even in early stages—within three months of infection—such as fine ureteral calcification that appears as a line or two parallel lines on conventional abdominopelvic X-ray, or as a circular pattern on axial CT images, which are pathognomonic for early-stage Schistosomiasis. Coarse calcification, fibrosis, and strictures are signs of late-stage Schistosomiasis. *Schistosoma* calcification can occur in the genital organs, most prominently in the seminal vesicles; in the prostate in males; and in the vulva, cervix, and perineum in females. *Shistosoma mansoni* and *haematobium* eggs can reach the spinal cord, resulting in acute myelopathy with paraparesis, urinary retention, or paraplegia. Ultimately, radiological signs only give a suggestive diagnosis, and detecting *Schistosoma* eggs either in stool (*Shistosoma mansoni*) or in urine (*Shistosoma haematobium*) is the gold standard diagnostic test.

## Data Availability

The available data are used in this manuscript.

## Abbreviations

CT	Computed Tomography
MRI	Magnetic Resonance Imaging
WHO	World Health Organization
CNS	Central Nervous System
POC-CCA	The Point-Of-Care Circulating Cathodic Antigen
NAATs	Nucleic Acid Amplification Tests
PCR	Polymerase Chain Reaction
cm	Centimeter

## References

[1] Singer B.J., Gomes M., Coulibaly J.T., Daigavane M., Tan S.T., Bogoch I.I., Lo N.C. (2025). Population-level impact of mass drug administration against schistosomiasis with anthelmintic drugs targeting juvenile schistosomes: A modelling study. Lancet Microbe.

[2] Caldwell N., Afshar R., Baragaña B., Bustinduy A.L., Caffrey C.R., Collins J.J., Fusco D., Garba A., Gardner M., Gomes M. (2023). Perspective on Schistosomiasis Drug Discovery: Highlights from a Schistosomiasis Drug Discovery Workshop at Wellcome Collection, London, September 2022. ACS Infect. Dis..

[3] Ally O., Kanoi B.N., Ochola L., Nyanjom S.G., Shiluli C., Misinzo G., Gitaka J. (2024). Schistosomiasis diagnosis: Challenges and opportunities for elimination. PLoS Negl. Trop. Dis..

[4] Ponzo E., Midiri A., Manno A., Pastorello M., Biondo C., Mancuso G. (2024). Insights into the epidemiology, pathogenesis, and differential diagnosis of schistosomiasis. Eur. J. Microbiol. Immunol..

[5] McManus D.P., Dunne D.W., Sacko M., Utzinger J., Vennervald B.J., Zhou X. (2018). Schistosomiasis. Nat. Rev. Dis. Primers.

[6] Olveda D.U., Olveda R.M., Lam A.K., Chau T.N., Li Y., Gisparil A.D., Ross A.G. (2014). Utility of Diagnostic Imaging in the Diagnosis and Management of Schistosomiasis. Clin. Microbiol..

[7] Cozzi D., Bertelli E., Savi E., Verna S., Zammarchi L., Tilli M., Rinaldi F., Pradella S., Agostini S., Miele V. (2020). Ultrasound findings in urogenital schistosomiasis: A pictorial essay. J. Ultrasound.

[8] Alsaedi H.I., Krsoom A.M., Alshoabi S.A., Alsharif W.M. (2022). Investigation Study of Ultrasound Practitioners’ Awareness about Artefacts of Hepatobiliary Imaging in Almadinah Almunawwarah. Pak. J. Med. Sci..

[9] Cimini A., Ricci M., Gigliotti P.E., Pugliese L., Chiaravalloti A., Danieli R., Schillaci O. (2021). Medical Imaging in the Diagnosis of Schistosomiasis: A Review. Pathogens.

[10] Nation C.S., Da’dara A.A., Marchant J.K., Skelly P.J. (2020). Schistosome migration in the definitive host. PLoS Negl. Trop. Dis..

[11] Richter J., da Costa C.P., Spangenberg T. (2026). The Parasite’s Life Cycle and the Patient. Praziquantel.

[12] Formenti F., Cortés A., Deiana M., Salter S., Parkhill J., Berriman M., Rinaldi G., Cantacessi C. (2023). The Human Blood Fluke, Schistosoma mansoni, Harbors Bacteria Throughout the Parasite’s Life Cycle. J. Infect. Dis..

[13] Llanwarne F., Helmby H. (2021). Granuloma formation and tissue pathology in Schistosoma japonicum versus Schistosoma mansoni infections. Parasite Immunol..

[14] Santos L.L., Santos J., Gouveia M.J., Bernardo C., Lopes C., Rinaldi G., Brindley P.J., Costa J.M.C.D. (2021). Urogenital Schistosomiasis-History, Pathogenesis, and Bladder Cancer. J. Clin. Med..

[15] Miller K., Choudry J., Mahmoud E.S., Lodh N. (2024). Accurate Diagnosis of Schistosoma mansoni and S. haematobium from Filtered Urine Samples Collected in Tanzania, Africa. Pathogens.

[16] Vaillant M.T., Philippy F., Neven A., Barré J., Bulaev D., Olliaro P.L., Utzinger J., Keiser J., Garba A.T. (2024). Diagnostic tests for human Schistosoma mansoni and Schistosoma haematobium infection: A systematic review and meta-analysis. Lancet Microbe.

[17] Hoekstra P.T., van Dam G.J., van Lieshout L. (2021). Context-Specific Procedures for the Diagnosis of Human Schistosomiasis—A Mini Review. Front. Trop. Dis..

[18] Dessein H., Duflot N., Romano A., Opio C., Pereira V., Mola C., Kabaterene N., Coutinho A., Dessein A. (2020). Genetic algorithms identify individuals with high risk of severe liver disease caused by schistosomes. Hum. Genet..

[19] Elbaz T., Esmat G. (2013). Hepatic and intestinal schistosomiasis: Review. J. Adv. Res..

[20] Andrianah G.E.P., Rakotomena D., Rakotondrainibe A., Rajaonarison Ny Ony L.H.N., Ranoharison H.D., Ratsimba H.R., Rajaonera T., Ahmad A. (2020). Contribution of Ultrasonography in the Diagnosis of Periportal Fibrosis Caused by Schistosomiasis. J. Med. Ultrasound.

[21] Lim M.C., Quek E.J.W., Chan T.Y.H., Teng M., Ng C.H., Gnanavelou M., Chen V.L., Fallowfield J.A., Zhang H., Noureddin M. (2026). Systematic Review: Imaging-Based Morphological Criteria for Liver Cirrhosis-A Call to Standardise. Aliment. Pharmacol. Ther..

[22] Liu J., Lu P., Dong C., Yang G., Zhou Y., Jia N., Li H. (2025). The Imaging Manifestations of Liver Cirrhosis. Radiology of Hepatobiliary Diseases.

[23] Chou E., Gadani S., Liu X. (2025). Noninvasive and Invasive Methods for the Diagnosis of Portal Hypertension. Tech. Vasc. Interv. Radiol..

[24] Strickland G.T. (1994). Gastrointestinal manifestations of schistosomiasis. Gut.

[25] Emara M.H., Mahros A.M., Rasheda A.M.A., Radwan M.I., Mohamed B., Abdelrazik O., Elazab M., Elbatae H. (2023). Schistosomal (bilharzial) polyps: Travel through the colon and beyond. World J. Gastroenterol..

[26] Akhtar M.M., ALJuhani N., Younus D., Alsahafi A.H., Abouhamda A. (2020). Schistosomiasis Mansoni Manifesting as Multiple Colonic Polyps. Cureus.

[27] Elhoseeny M.M., Sallam A., Eladl A.E., Othman A.A.A. (2026). Bleeding rectal polyp as an atypical presentation of intestinal schistosomiasis: A case report from Egypt. PLoS Negl. Trop. Dis..

[28] Shebel H.M., Elsayes K.M., Abou El Atta H.M., Elguindy Y.M., El-Diasty T.A. (2012). Genitourinary schistosomiasis: Life cycle and radiologic-pathologic findings. RadioGraphics.

[29] Lee H.J., Sung W.S. (2018). Calcification of the urinary bladder and ureter in schistosomiasis. Kidney Res. Clin. Pract..

[30] Lackey E.K., Horrall S. (2026). Schistosomiasis. StatPearls.

[31] El Boté H., Boughaleb A. (2025). Tuberculosis of seminal vesicles: A rare case report. IDCases.

[32] Ladumor H., Al-Mohannadi S., Ameerudeen F.S., Ladumor S., Fadl S. (2021). TB or not TB: A comprehensive review of imaging manifestations of abdominal tuberculosis and its mimics. Clin. Imaging.

[33] Alshoabi S.A., Almas K.M., Aldofri S.A., Hamid A.M., Alhazmi F.H., Alsharif W.M., Abdulaal O.M., Qurashi A.A., Aloufi K.M., Alsultan K.D. (2022). The Diagnostic Deceiver: Radiological Pictorial Review of Tuberculosis. Diagnostics.

[34] Cheng P.Y., Huang Y.Y., Jaw F.S., Chung S.D., Tsai C.Y. (2022). Diffused bladder wall calcification in a survivor with severe coronavirus disease 2019: A case report. Medicine.

[35] Orish V.N., Morhe E.K.S., Azanu W., Alhassan R.K., Gyapong M. (2022). The parasitology of female genital schistosomiasis. Curr. Res. Parasitol. Vector Borne Dis..

[36] de Wilton A., Aggarwal D., Jäger H.R., Manji H., Chiodini P.L. (2021). Delayed diagnosis of spinal cord schistosomiasis in a non-endemic country: A tertiary referral centre experience. PLoS Negl. Trop. Dis..

[37] Ferrari T.C., Moreira P.R. (2011). Neuroschistosomiasis: Clinical symptoms and pathogenesis. Lancet Neurol..

[38] Alanazi R.F., Al Karawi M., Almalki A., Sufiani F., Al Karawi S. (2023). Schistosomiasis Involving the Central Nervous System: Case Report of a Rare Complication. Case Rep. Surg..

[39] Arega G., Adane L., Mekonnen E., Negussie M.A. (2025). Spinal schistosomiasis masquerading as spinal cord tumor in a 12-year-old male adolescent: A case report. Radiol. Case Rep..

